# Visible photoassisted room-temperature oxidizing gas-sensing behavior of Sn_2_S_3_ semiconductor sheets through facile thermal annealing

**DOI:** 10.1186/s11671-016-1720-2

**Published:** 2016-11-16

**Authors:** Yuan-Chang Liang, Tsai-Wen Lung, Chein-Chung Wang

**Affiliations:** Institute of Materials Engineering, National Taiwan Ocean University, Keelung, 20224 Taiwan

**Keywords:** Semiconductor, Sheets, Crystal feature, Surface, Gas-sensing response

## Abstract

Well-crystallized Sn_2_S_3_ semiconductor thin films with a highly (111)-crystallographic orientation were grown using RF sputtering. The surface morphology of the Sn_2_S_3_ thin films exhibited a sheet-like feature. The Sn_2_S_3_ crystallites with a sheet-like surface had a sharp periphery with a thickness in a nanoscale size, and the crystallite size ranged from approximately 150 to 300 nm. Postannealing the as-synthesized Sn_2_S_3_ thin films further in ambient air at 400 °C engendered roughened and oxidized surfaces on the Sn_2_S_3_ thin films. Transmission electron microscopy analysis revealed that the surfaces of the Sn_2_S_3_ thin films transformed into a SnO_2_ phase, and well-layered Sn_2_S_3_–SnO_2_ heterostructure thin films were thus formed. The Sn_2_S_3_–SnO_2_ heterostructure thin film exhibited a visible photoassisted room-temperature gas-sensing behavior toward low concentrations of NO_2_ gases (0.2–2.5 ppm). By contrast, the pure Sn_2_S_3_ thin film exhibited an unapparent room-temperature NO_2_ gas-sensing behavior under illumination. The suitable band alignment at the interface of the Sn_2_S_3_–SnO_2_ heterostructure thin film and rough surface features might explain the visible photoassisted room-temperature NO_2_ gas-sensing responses of the heterostructure thin film on exposure to NO_2_ gas at low concentrations in this work.

## Background

Binary tin sulfide semiconductors, because of their narrow band gap values and *n*-type semiconducting characteristics, have attracted considerable attention in applications of various photovoltaic and photoactivated devices [1–4]. For practical scientific applications, tin sulfide semiconductors in the form of a thin-film structure are in high demand. Several synthesis techniques, such as spray pyrolysis [3], thermal evaporation [4], chemical bath deposition [5], and chemical vapor deposition [6], have been successfully employed in preparing tin sulfide semiconductor thin films. Sputtering thin-film preparation methods are frequently used to fabricate complex compounds in the semiconductor industry because they enable easy process control, large area deposition, feasibility with a wide range of thin-film thickness, and high reproducibility [7–9]. However, few reports on thin-film synthesis of tin sulfide semiconductors through sputtering techniques have been published.

Resistive gas sensors are crucial in industrial and medical applications for detecting pollutants as well as toxic and combustible gases. Growing interest has inspired the development of diverse gas sensors used at low temperatures or in room-temperature environments [10]. However, overcoming high barriers is generally necessary for the gas molecules to adsorb onto and desorb from the semiconductor surfaces during gas-sensing response and recovery at low temperatures; therefore, fabricating thin-film semiconductor-based gas sensors that have visible gas-sensing behaviors at room temperature remains a considerable challenge. Moreover, narrow band gap tin sulfide semiconductors have rarely been reported for applications in gas-sensing materials operated at room temperature. Recently, various heterostructures have been widely investigated and show enhanced functions of the single constituent counterpart [11–13]. Moreover, a novel concept of sensing mechanisms, such as gas sensing based on the variation in the photoelectric response, was proposed [14]. Photoexcited carriers were shown to be crucial for improving the gas-sensing responses of semiconductor-based sensors operated at a low room temperature [15]. Several heterostructure systems such as TiO_2_–CdS and SnO_2_–ZnO exhibit clear reducing and oxidizing gas-sensing properties, respectively, under light illumination at room temperature [16, 17]. In the present study, tin sulfide thin films with a sheet-like surface were synthesized through radiofrequency (RF) sputtering. The as-synthesized tin sulfide thin films exhibited a visible gas-sensing response to low-concentration NO_2_ gases under light illumination at room temperature when the films were subjected to a simple postthermal annealing procedure. The correlation between the tin sulfide thin film microstructures and oxidizing gas-sensing properties was initially investigated in this study.

## Methods

In this study, the preparation of Sn_2_S_3_–SnO_2_ heterostructure thin film consists of two steps. First, Sn_2_S_3_ thin films were fabricated onto 300-nm-thick SiO_2_/Si substrates using RF magnetron sputtering. SnS_2_ ceramics target was used to sputtering growth Sn_2_S_3_ thin film which has a nonstoichiometric composition from the target material. During the growth of Sn_2_S_3_ thin film, the sputtering power of SnS_2_ target was fixed at 40 W. The thin-film growth temperature of the Sn_2_S_3_ thin films was maintained at 250 °C in pure Ar ambient; the gas pressure during the sputtering thin-film deposition was fixed at 0.67 Pa in this work. Subsequently, the as-grown Sn_2_S_3_ thin films were subjected to a postannealing procedure at 400 °C for 40 min in ambient air. This process engendered the surfaces of the Sn_2_S_3_ thin films oxidized and form the thin SnO_2_ crystallites on the surfaces of the Sn_2_S_3_ thin films.

Thin-film crystal structures were investigated by X-ray diffraction (XRD; Bruker D2 PHASER) using Cu Kα radiation with a theta-two theta scan mode. The surface morphology of the samples was investigated by scanning electron microscopy (SEM; Hitachi S-4800). The detailed microstructures of the as-synthesized samples were characterized by high-resolution transmission electron microscopy (HRTEM; Philips Tecnai F20 G2). The composition analysis was performed using an energy-dispersive X-ray spectrometer (EDS) attached to the TEM. To measure NO_2_ gas-sensing properties of the Sn_2_S_3_ and Sn_2_S_3_–SnO_2_ thin films, the Pt interdigital electrodes were patterned onto the substrates by DC sputtering. Subsequently, the Sn_2_S_3_ and Sn_2_S_3_–SnO_2_ thin films were prepared onto the Pt electrodes-coated substrates to form gas sensor devices. The gas-sensing measurements of the sensor were conducted at 5 V. A 100-W Xe arc lamp was used as the illumination source for light-assisted gas-sensing tests. The gas-sensing tests were conducted with various concentrations of NO_2_ gas (0.2, 0.5, 1.0, and 2.5 ppm) at room temperature. The gas-sensing response of the sensor to NO_2_ gas is defined as the Rg/Ra. Ra is the sensor electrical resistance in the absence of target gas and Rg is that in the target gas. The gas-sensing response of the sensor to 100 ppm CO, H_2_, and NH_3_ gases is defined as the Ra/Rg.

## Results and Discussion

**Fig. 1:**
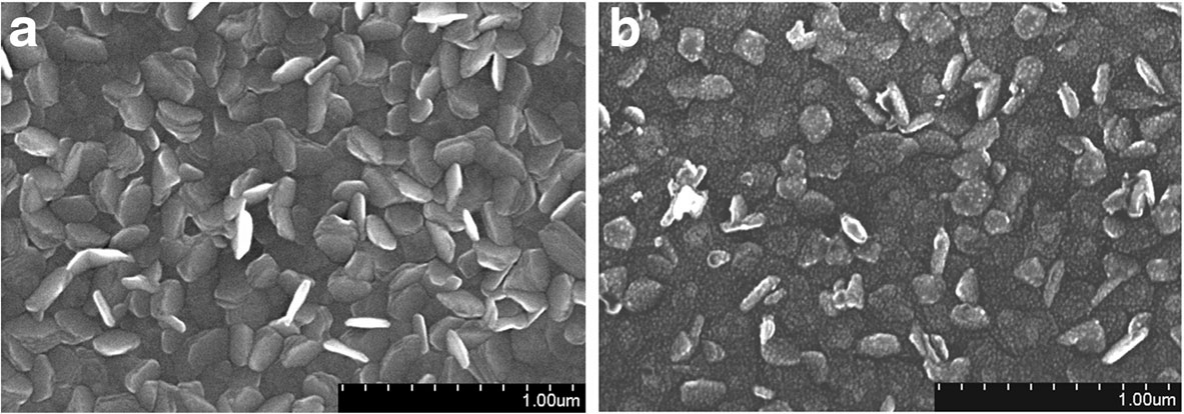
SEM images. **a** Sn_2_S_3_ thin film. **b** Sn_2_S_3_–SnO_2_ thin film

**Fig. 2:**
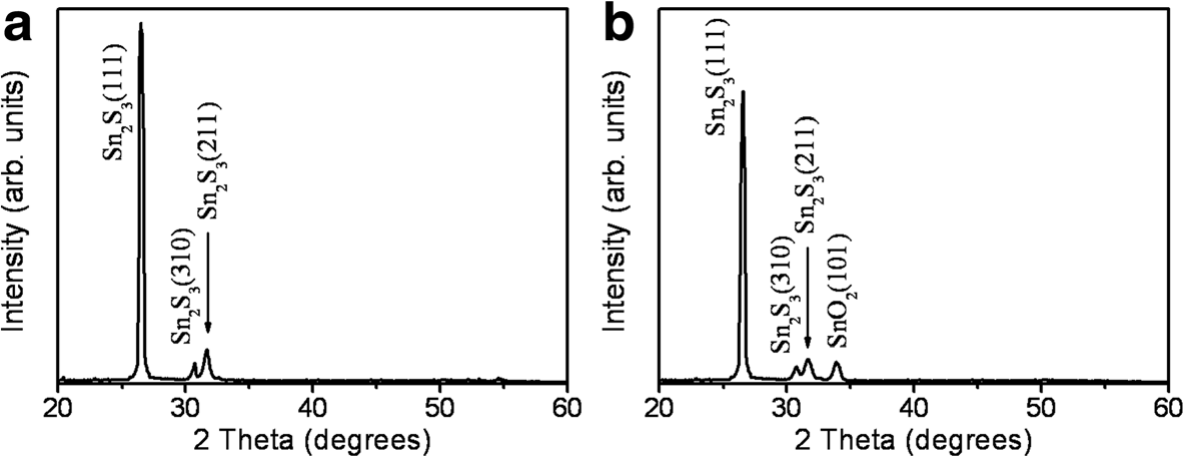
XRD patterns. **a** Sn_2_S_3_ thin film. **b** Sn_2_S_3_–SnO_2_ thin film

Figure 1a, b shows the morphologies of the sputtering-deposited Sn_2_S_3_ thin films with and without postannealing in ambient air. Fig. 1a shows that the surface of the Sn_2_S_3_ film has a visible sheet-like texture. These sheet-like crystallites had a sharp periphery and homogeneously covered the film surface. The size of the sheet-like surface crystallites ranged 150–300 nm. A similar sheet-like surface morphology was also reported for Sn_2_S_3_ thin films synthesized using a chemical solution method [5]. The periphery of the sheet-like Sn_2_S_3_ became rough and passivated after the postannealing procedure. Such a transformation in the morphology of a solid thin-film surface after various postannealing procedures has been attributed to factors such as the phase changes, the composition changes, or the growth of the grain size during postannealing procedures [18, 19]. Figure 2a, b shows XRD patterns of the Sn_2_S_3_ thin film with and without a postannealing procedure. Figure 1c shows three marked Bragg reflections centered at approximately 26.5°, 30.7°, and 31.7°. These Bragg reflections originated from the (111), (310), and (211) crystallographic planes of the orthorhombic Sn_2_S_3_ (JCPDS No. 14-0619). The XRD result indicated that the Sn_2_S_3_ thin film mainly consisted of highly (111)-oriented grains. The XRD pattern showed that when the Sn_2_S_3_ thin film was postannealed in ambient air, it still exhibited a highly (111)-preferential orientation. Moreover, a visible Bragg reflection of SnO_2_ (101) appears in Fig. 2b (JCPDS No. 88-0287), confirming the formation of Sn_2_S_3_–SnO_2_ heterostructure thin film with a high crystallinity.

**Fig. 3:**
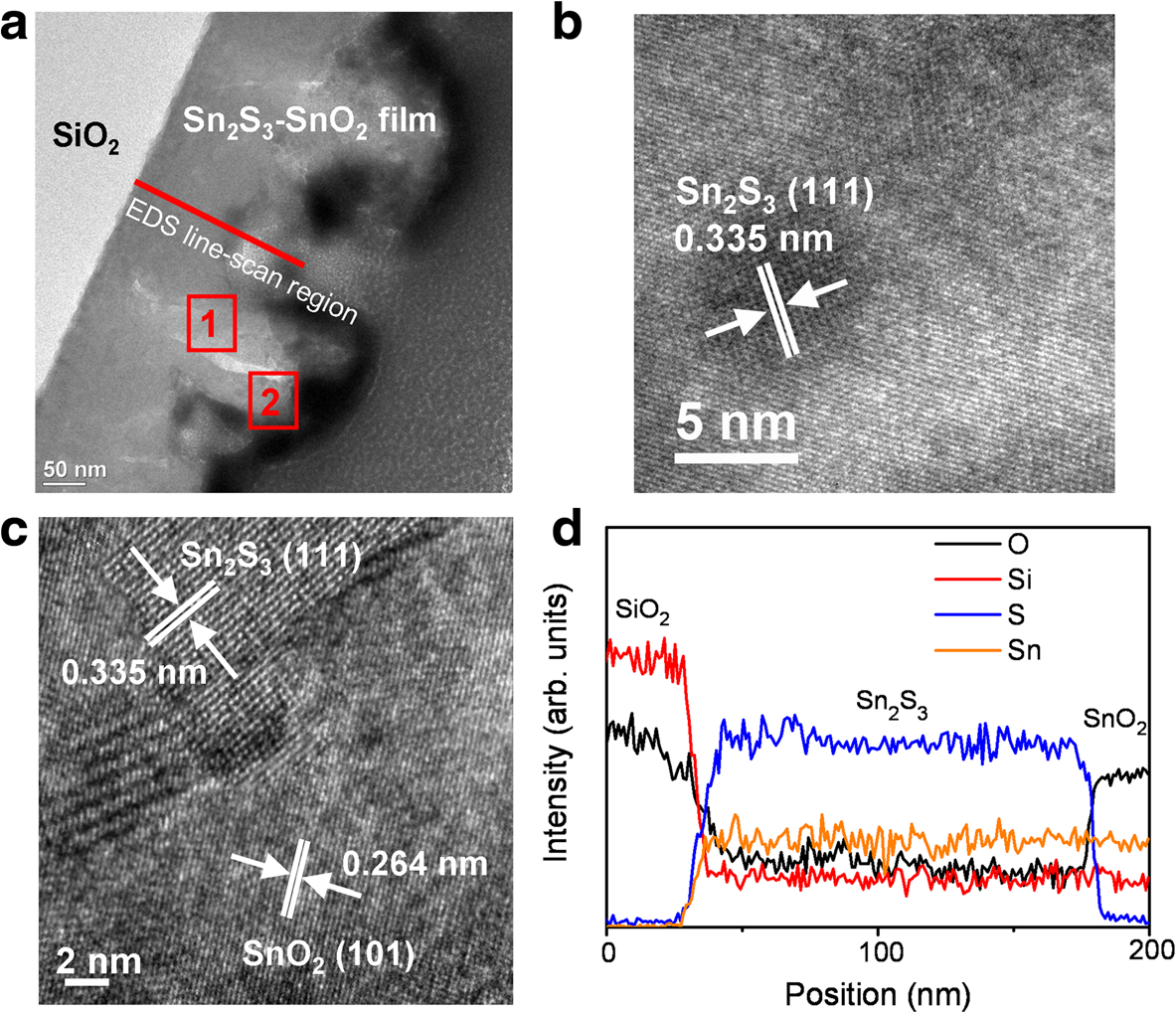
TEM analyses of the Sn_2_S_3_–SnO_2_ thin film. **a** Low-magnification cross-sectional TEM image of the film. **b** HRTEM image taken from the inner region of the film (marked with *1* in **a**). The region corresponded to the region of Sn_2_S_3_ phase. **c** HRTEM image taken from the outer region of the film (marked with *2* in **a**). It shows that the SnO_2_ phase distributed on the outer region of the Sn_2_S_3_ phase. **d** Cross-sectional EDS line-scan profiling spectra of Si, Sn, S, and O elements taken from the film (as shown with a *red line* in **a**)

Figure 3a shows a low-magnification cross-sectional TEM image of the Sn_2_S_3_–SnO_2_ heterostructure thin film. The TEM image revealed that the film had an undulated surface morphology. The surface of the film was considerably rugged. The drop height of the surface protrusions evaluated from the TEM image ranged approximately 100–150 nm. Figure 3b shows a HRTEM image taken from the internal region of the Sn_2_S_3_–SnO_2_ heterostructure (region 1 marked in Fig. 2a). Ordered and clear lattice fringes were observed in the HRTEM image, revealing that the film had a high crystallinity. Moreover, the local lattice fringes with an interval of approximately 0.335 nm corresponded to the interatomic distance of orthorhombic Sn_2_S_3_ (111). The HRTEM image taken from the outer region (region 2 marked in Fig. 3a) of the film is shown in Fig. 3c. Clear and ordered lattice fringes with intervals of approximately 0.335 nm corresponded to orthorhombic Sn_2_S_3_ (111), and the lattice fringes with an interval of approximately 0.264 nm in the outer region matched the spacing distance of SnO_2_ (101). To further confirm the favorable formation of the Sn_2_S_3_–SnO_2_ layered heterostructure through the annealing of the Sn_2_S_3_ thin film in ambient air, the spatial distributions of the atomic composition across the Sn_2_S_3_–SnO_2_ film were obtained using a nanoprobe EDS line-scan profiling analysis (Fig. 3d). The Sn signal was homogeneously distributed over the measured region. By contrast, an O signal and an S signal with a clear intensity drop were observed in the outer region of the film, showing that the outer region of the film was mainly composed of Sn and O elements, and the internal region of the film consisted of Sn and S elements. The TEM analyses revealed that a well-layered Sn_2_S_3_–SnO_2_ heterostructure film was formed when the Sn_2_S_3_ film was treated using the described postannealing procedure in ambient air.

**Fig. 4:**
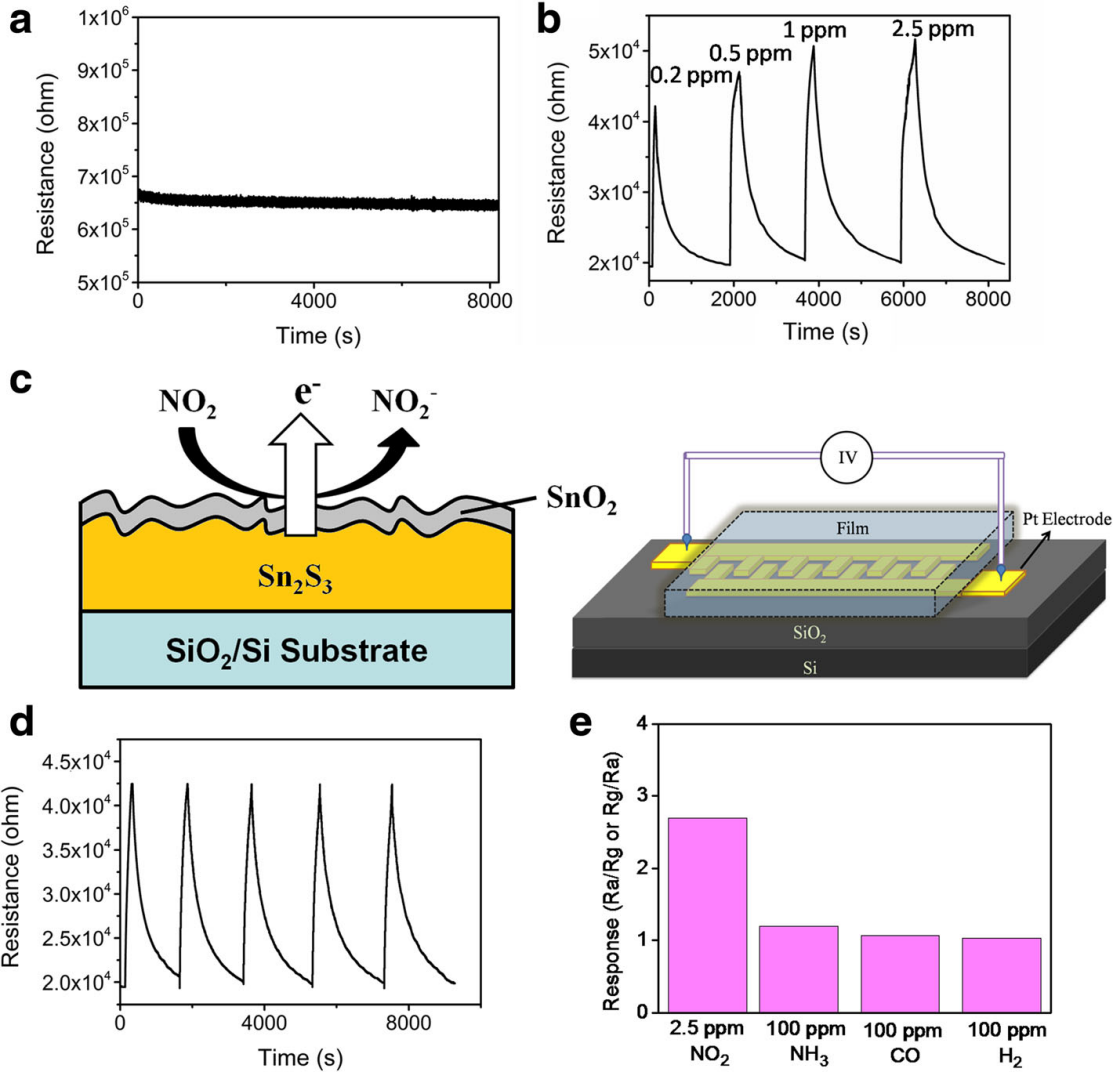
**a** Dynamic electrical resistance variation curves of the Sn_2_S_3_ thin film on exposure to various NO_2_ gas concentrations (0.2–2.5 ppm) under light irradiation. **b** Dynamic electrical resistance variation curves of the Sn_2_S_3_–SnO_2_ thin film on exposure to various NO_2_ gas concentrations (0.2–2.5 ppm) under light irradiation. **c** Schematics of the Sn_2_S_3_–SnO_2_ thin film surface reaction with NO_2_ gas molecules and gas sensor device. **d** Cyclic gas-sensing response curves of the Sn_2_S_3_–SnO_2_ thin film on exposure to 0.2 ppm NO_2_ gas under light irradiation. **e** The gas-sensing responses of the Sn_2_S_3_–SnO_2_ thin film on exposure to various test gases

**Fig. 5:**
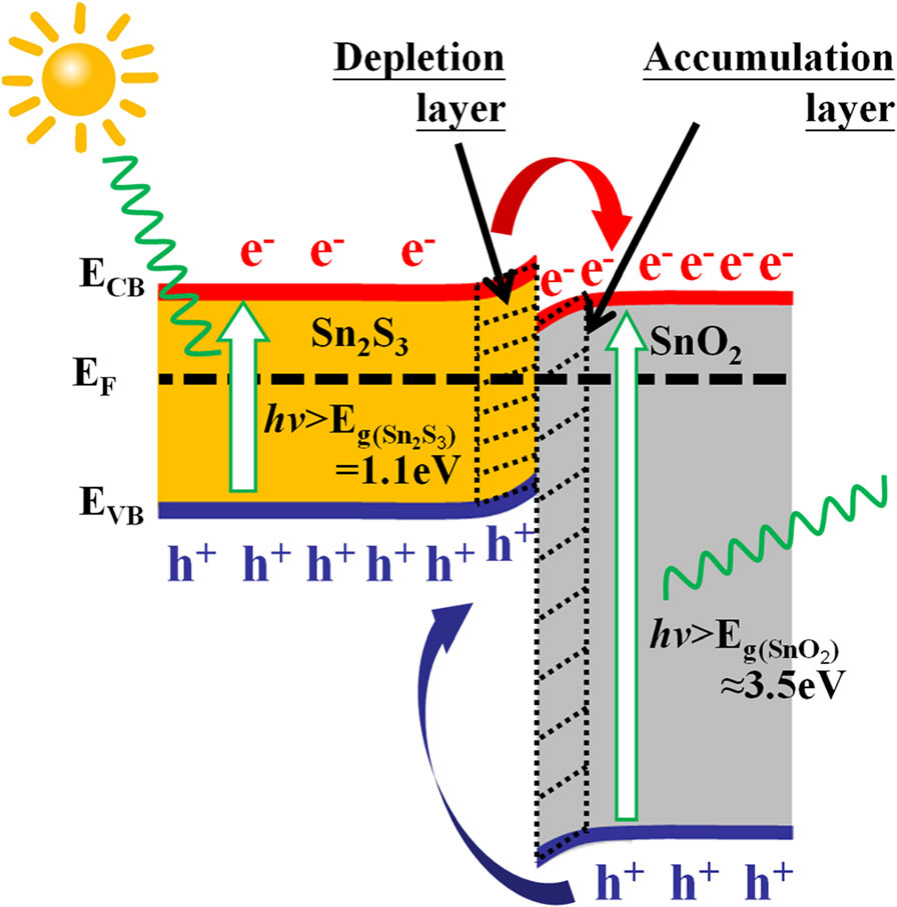
Band alignment of the Sn_2_S_3_–SnO_2_ thin film under light irradiation

The photoassisted gas-sensing properties of the Sn_2_S_3_ film with and without postannealing were measured by recording the change in electrical resistance on exposure to various NO_2_ gas concentrations. Figure 4a, b shows the dynamic electrical resistance variations of the Sn_2_S_3_ film and Sn_2_S_3_–SnO_2_ heterostructure film on exposure to 0.2–2.5 ppm of NO_2_ gases under light illumination at room temperature. The electrical resistance of the *n*-type Sn_2_S_3_ film under light illumination was nearly unchanged and maintained at an electrical resistance value of approximately 660 kΩ on exposure to the various NO_2_ gas concentrations. The Sn_2_S_3_ film in this work was inactive when detection of oxidizing NO_2_ gas under light illumination at room temperature was attempted. The possible reasons for the inactive gas-sensing responses of the Sn_2_S_3_ film under light irradiation might be associated with the sensor operating at room temperature, and the measuring system did not provide sufficient thermal energy for NO_2_ gas molecules to overcome the barriers to adsorb onto the surface of the Sn_2_S_3_ during the gas-sensing test. Moreover, a high recombination rate of photoexcited carriers might have occurred in the Sn_2_S_3_ crystallites, which would not have efficiently provided sufficient free electrons on the surfaces of the sheet-like Sn_2_S_3_ crystallites under light illumination. These factors might account for the observed lack of clear oxidizing gas-sensing responses of the sheet-like Sn_2_S_3_ film under light irradiation at room temperature. By contrast, a clear gas-sensing behavior was observed for the Sn_2_S_3_–SnO_2_ heterostructure thin film toward various NO_2_ gas concentrations (Fig. 4b). Schematics of NO_2_ gas-sensing reaction on the surface of the heterostructure thin film and gas sensor device are illustrated in Fig. 4c. The initial electrical resistance of the Sn_2_S_3_–SnO_2_ heterostructure thin film under illumination was approximately 19 kΩ. When NO_2_ gas was introduced into the test chamber, the measured electrical resistance value of the heterostructure thin film showed a visible increase. Moreover, the cyclic gas-sensing response test for the Sn_2_S_3_–SnO_2_ heterostructure thin film on exposure to 0.2 ppm NO_2_ was conducted five times (Fig. 4d). The high repeatability of the gas-sensing response curves revealed that the Sn_2_S_3_–SnO_2_ heterostructure thin film is highly reliable for detecting NO_2_ gas under light illumination at room temperature. Notably, the gas-sensing responses of the Sn_2_S_3_–SnO_2_ heterostructure thin film are inactive on exposure to other test gases under light illumination at room temperature (Fig. 4e). The substantial improvement of the oxidizing gas-sensing responses of the Sn_2_S_3_ film with a moderate postannealing procedure is attributable to the energy band structure of the Sn_2_S_3_–SnO_2_ heterostructure. The work function of Sn_2_S_3_ is approximately 4.13 eV and that of SnO_2_ is approximately 4.9 eV [20, 21]. The work function of Sn_2_S_3_ is smaller than that of SnO_2_; it can be assumed that the interface of SnO_2_–Sn_2_S_3_ is crucial to improving the spatial separation of photoexcited carriers under light illumination. A schematic of the energy-band structure of the Sn_2_S_3_–SnO_2_ heterostructure thin film is illustrated in Fig. 5. A noticeable photoexcited charge transfer between the Sn_2_S_3_ and SnO_2_ was expected. The efficient spatial charge separation prolonged the lifetime of the charges and might have increased the electron density on the surfaces of the sheet-like Sn_2_S_3_–SnO_2_ heterostructure thin film [22]. The formation of the marked electron accumulation layer on the SnO_2_ side of the SnO_2_–Sn_2_S_3_ heterostructure was crucial to observing the enhanced gas-sensing properties of the Sn_2_S_3_–SnO_2_ heterostructure in this work. During the gas-sensing process, the electrical resistance increase can be associated with surface-controlled processes and explained by the capturing of free electrons from the surfaces of the *n*-type semiconductors by adsorbed oxidizing NO_2_ gas molecules [23, 24]. Such adsorbed NO_2_ gas molecules could capture the electrons from the SnO_2_ surface of the heterostructure thin film and might have engendered the variation of the depletion layer thickness at the heterointerfaces, resulting in the increased electrical resistance of the sample. This indicated that the higher concentration of electrons on the surfaces of the Sn_2_S_3_–SnO_2_ heterostructure thin film compared with that on the Sn_2_S_3_ thin film surface can yield a greater opportunity for reaction with NO_2_ gas molecules under light illumination at room temperature. Furthermore, the rugged surface of the sheet-like surface morphology of the Sn_2_S_3_–SnO_2_ thin film is more favorable for NO_2_ gas molecules adsorption than that of the Sn_2_S_3_ film. A marked electrical resistance variation of the Sn_2_S_3_–SnO_2_ heterostructure thin film during the oxidizing gas-sensing tests was expected. Notably, the enhancement of gas-sensing properties of the CdS nanowires under light illumination at room temperature was demonstrated in a previous study through the formation of CdS–ZnO heterostructures; the effective interfacial transport of excess carriers contributed to the improved gas-sensing responses [25]. Moreover, in another study, rod-like WO_3_-based heterostructures demonstrated improved conductivity, specific electron transfer, and increased gas adsorption compared with those of the single constituent counterpart; these factors contribute to their enhanced light-driven gas-sensing responses to NO_2_ gas [26]. Therefore, the suitable band structure of the Sn_2_S_3_–SnO_2_ heterostructure thin film and a rugged surface contributed to the substantial photoassisted room-temperature NO_2_ gas-sensing responses of the Sn_2_S_3_–SnO_2_ heterostructure thin film on exposure to low concentrations of NO_2_ gases.

## Conclusions

In summary, highly (111)-oriented Sn_2_S_3_ semiconductor thin films with a sheet-like surface were grown through RF sputtering. The as-synthesized Sn_2_S_3_ semiconductor thin films were subsequently treated using postthermal annealing in ambient air at 400 °C. SEM analysis revealed that the surfaces of the Sn_2_S_3_ thin films were roughened after postannealing in ambient air. Both XRD and TEM analyses indicated that the Sn_2_S_3_–SnO_2_ heterostructure thin films were formed through the formation of the SnO_2_ phase on the surfaces of the Sn_2_S_3_ thin films. By contrast, the Sn_2_S_3_–SnO_2_ heterostructure thin films exhibited visible photoassisted room-temperature gas-sensing responses to low NO_2_ gas concentrations. The substantially improved photoassisted room-temperature NO_2_ gas-sensing responses of the Sn_2_S_3_ thin films through facile thermal annealing is attributable to the increased surface roughness and enhanced spatial carrier separation efficiency of the Sn_2_S_3_–SnO_2_ heterostructure thin films.

## Abbreviations

EDS: Energy-dispersive X-ray spectrometer
HRTEM: High-resolution transmission electron microscopy
SEM: Scanning electron microscopy
XRD: X-ray diffraction
